# The SP-ET index is a new index for assessing the vertical position of patella

**DOI:** 10.1186/s13244-022-01289-2

**Published:** 2022-09-24

**Authors:** Jia Li, Mao Yuan, Lanyu Qiu, Bo Sheng, Fan Yu, Haitao Yang, Furong Lv, Fajin Lv, Wei Huang

**Affiliations:** 1 Department of Radiology, The First Affiliated Hospital of Chongqing Medical University, Chongqing, People’s Republic of China; 2 Department of Orthopaedic, The First Affiliated Hospital of Chongqing Medical University, 1 Youyi Road, Yuzhong District, Chongqing, 400016 People’s Republic of China; 3 Orthopaedic Research Laboratory, Chongqing Medical University, Chongqing, 400016 People’s Republic of China

**Keywords:** Patellar cartilage lesions, SP-ET index, Insall–Salvati ratio, Patellofemoral joint, Vertical position

## Abstract

**Background:**

Some parameters in previous studies did not better reflect the vertical position of the patella relative to the femoral trochlear. This study aimed to assess the value of the most superior point of patella-entrance of femoral trochlea distance ratio (SP-ET index) as a newer index in defining the vertical position of patella relative to the trochlea, correlate it with the Insall–Salvati ratio, and investigate the effect of the new index on patellar cartilage lesions.

**Methods:**

A total of 99 knees of 77 patients with patellar cartilage lesions were retrospectively analyzed using magnetic resonance imaging (MRI) data. The Insall–Salvati ratio and SP-ET index were measured on MR images. Ninety-nine knees just with meniscus rupture were enrolled as the control group. The two parameters of the patellar cartilage lesions were compared with those of the control group.

**Results:**

The Insall–Salvati ratio and SP-ET index in the patellar cartilage lesions group were significantly higher than those in the control group (*p* < 0.001). The SP-ET index showed a moderate positive correlation with the Insall–Salvati ratio (*r* = 0.307, *p* < 0.001). Receiver operating characteristic (ROC) analysis showed that the diagnostic efficiency of the SP-ET index was better than that of the Insall–Salvati ratio in patients with patellar cartilage lesions.

**Conclusion:**

The SP-ET index may be a useful complement parameter to define the vertical position of the patella relative to the femoral trochlear. Increased SP-ET index may be an important risk factor for patellar cartilage lesions.

## Key points

1. The Insall–Salvati ratio has limitations.
2. SP-ET index may be useful for defining the vertical position of patella.
3. Increased SP-ET index is an important risk factor for patellar cartilage lesions.

## Introduction

The primary anatomical factors which were associated with patellofemoral malalignment included patella alta, trochlear dysplasia, and abnormal lateral patellar tilt [1, 2]. Patella maltracking is often connected with rotational deformities of the lower extremity, including increased femoral anteversion, external tibial rotation, or genu valgum [3, 4]. In addition, imbalances in the strength of the muscles around the knee joint can also lead to patella maltracking [2, 5]. Patellofemoral malalignment resulted in abnormal joint contact and chondral stresses within the patellofemoral compartment and was often associated with chondral damage and chronic anterior knee pain [6]. Patellar cartilage lesion caused by various diseases, including osteoarthritis, as chondromalacia patella, is one of the common causes of chronic anterior knee pain and leads to a decrease in quality of life [7–9]. A progressive loss of articular cartilage can lead to the development of patellofemoral osteoarthritis [10, 11]. The most commonly cited hypothesis is patellofemoral malalignment and/or maltracking that leads to pathological loading on the patellofemoral joint and increases subsequent articular cartilage wear [8, 12], and we found that there have been several studies focusing on the relationship between patellofemoral malalignment and patellar cartilage lesions.

Duran et al. [13] and Tuna et al. [8] reported that there was an association between abnormal trochlear morphology and chondromalacia patella in women. In addition, the study of Lu et al. showed that the patellar height might be an important factor in the severity of patellar cartilage lesions [14]. Both patella alta and patella baja were found to be related to chondromalacia patella, and the modified Insall–Salvati index ratio was the best indicator to define patella alta [14, 15]. Patella alta may lead to excessive lateral motion of the patella during knee flexion [16] and cause the patella may not to be able to engage the trochlear groove early during knee flexion [17]; all of these could lead to the development of patellar cartilage lesions. Multiple methods of assessing the vertical position of the patella on imaging have been described in the previous literature [17, 18]. Most noteworthy, some studies suggested that the ratio between the articular cartilage of the patella and the trochlear cartilage parameters (patellotrochlear index [19] and patellar articular overlap [17]) could be better used to assess the vertical position of the patella in patients with patellofemoral instability.

In addition, there were some researches focusing on the role of trochlear dysplasia in chondromalacia patella or patellofemoral cartilage lesions [11, 13, 20], and the study of Ambra et al. [11] suggested that trochlear dysplasia might be more important than the patellar malposition in the pathogenesis of patellofemoral cartilage lesions. But the measurement parameters defining the vertical position of the patella in some studies did not better reflect the vertical position of the patella relative to the femoral trochlear as patellotrochlear index [19] and patellar articular overlap [17] do. Therefore, the present study aimed to identify the ratio of the most superior point of patella-entrance of femoral trochlea distance to the length of the patella articular surface on sagittal MRI as a newer index which can define the vertical position of patella relative to the trochlea and investigate the effect of this parameter on patellar cartilage lesions. We hypothesized that the new parameter was a good indicator to define the vertical position of the patella, and moreover, this increased parameter might be a potential risk factor for the development of patellar cartilage lesions.

## Materials and methods

### Participants

**Fig. 1:**
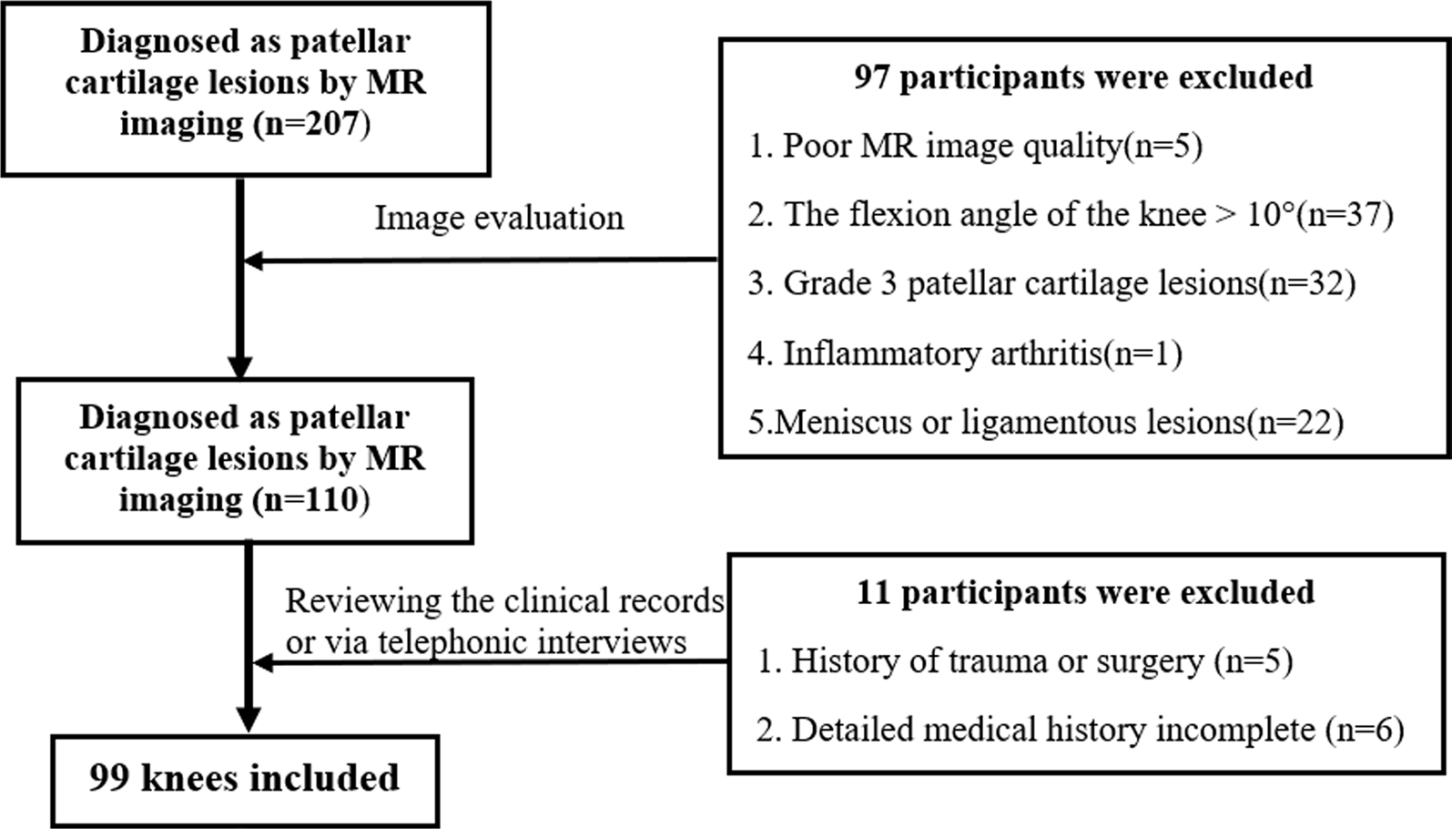
Flowchart for selection of participants in patellar cartilage lesions group

This retrospective study was approved by the Committee for Human Research of our institution (No. 2021-203). We retrospectively reviewed patients who were diagnosed with the modified Noyes classification system grade 1–2B patellar cartilage lesions based on MRI between January 2020 to April 2021 using picture archiving and communication systems at our department. Inclusion criteria were as follows: (1) patients with typical clinical presentation and MR signs of patellar cartilage lesions; (2) the flexion angle of the knee on MR images was 0°–10°. Exclusion criteria were as follows: (1) participants with a history of trauma or surgery of the knee; (2) presence of patellar dislocation, meniscus, or ligamentous lesions; (3) presence of severe osteoarthritis (≥ KL 3 grade) or inflammatory arthritis; and (4) poor MR image quality (Fig. 1). In order to obtain a control group of participants without anterior knee pain and without any detectable alignment abnormalities or structural lesions on MRI and arthroscopy, patients undergoing meniscus rupture repair were chosen. All the clinical information was acquired by reviewing the clinical records or collected by telephone for medical history if uncertain on records.

### MRI protocol

MRI was performed using 1.5-T MRI equipment (Magnetom Essenza; Siemens Healthcare) with an extremity matrix knee coil (a Tim coil). The imaging protocol was as follows: sagittal and coronal T1 turbo spin echo (repetition time/echo time 306/ 12 ms, field of view 16 cm, matrix size 320 × 320, slice thickness 4 mm), sagittal T2 turbo spin echo (repetition time/echo time 3220/99 ms, field of view 16 cm, matrix size 320 × 320, slice thickness 4 mm), sagittal and coronal intermediate-weighted turbo spin echo with fat saturation (repetition time/echo time 2800/38 ms, field of view 16 cm, matrix size 256 × 256, slice thickness 4 mm), and axial intermediate-weighted turbo spin echo with fat saturation (repetition time/echo time 2800/51 ms, field of view 16 cm, matrix size 232 × 256, slice thickness 4 mm).

### Image analyses

**Fig. 2:**
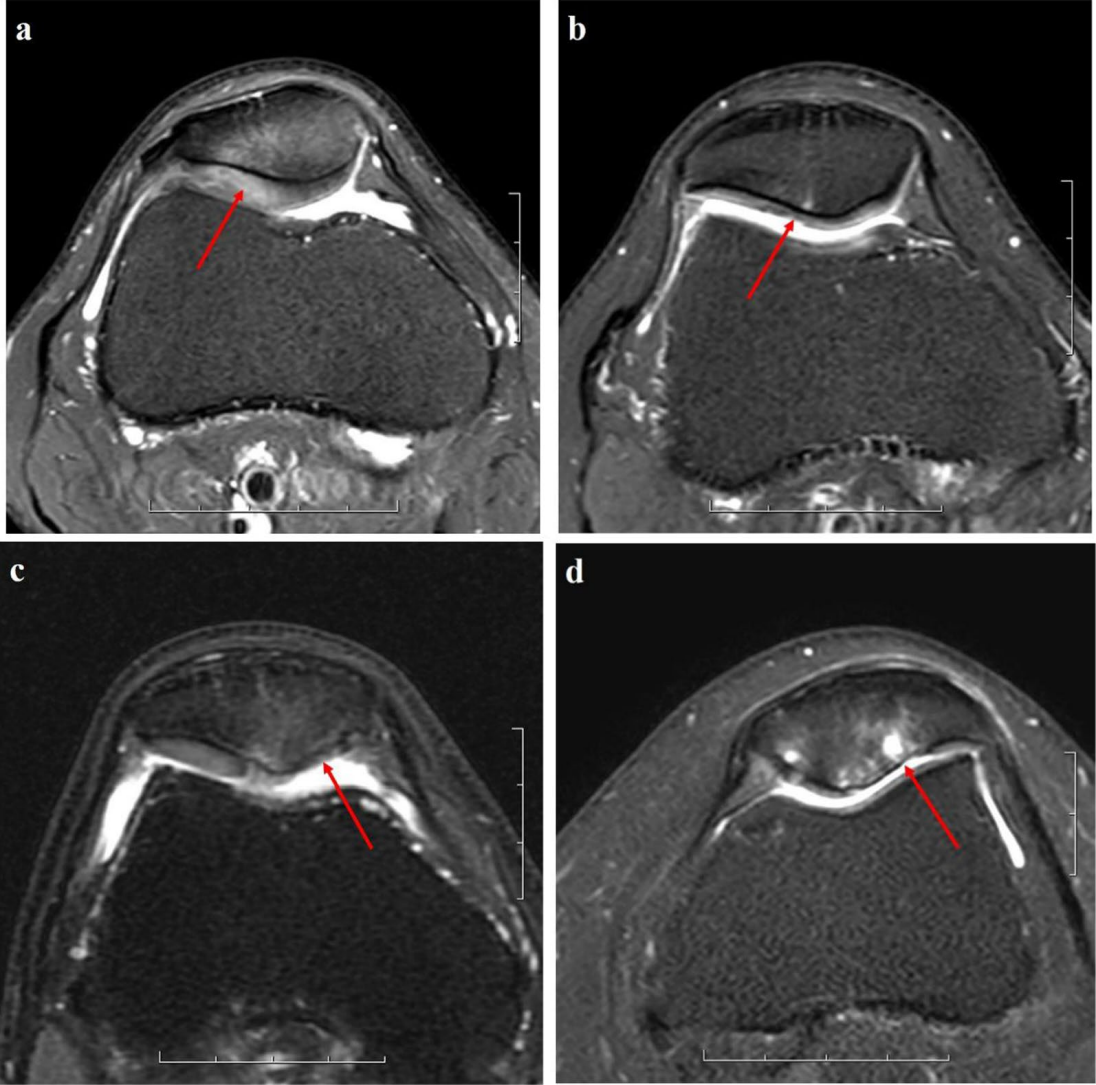
The modified Noyes classification system of patellar cartilage lesions. **a** Grade 1—increased T2 signal intensity of morphologically normal cartilage; **b** grade 2A—superficial partial-thickness cartilage defect less than 50% of total articular surface thickness; **c** grade 2B—deep partial-thickness cartilage defect greater than 50% of the total articular surface thickness; **d** grade 3—full-thickness cartilage defect

All MRI images were independently reviewed by two musculoskeletal radiologists (Qiu and Sheng, with 3 and 13 years of clinical experience, respectively). The routine MR imaging protocol was used to grade the articular cartilage on the patellofemoral joint according to the modified Noyes classification system: grade 0 = normal cartilage, grade 1 = increased T2 signal intensity of morphologically normal cartilage, grade 2A = superficial partial-thickness cartilage defect less than 50% of the total articular surface thickness, grade 2B = deep partial-thickness cartilage defect greater than 50% of the total articular surface thickness, and grade 3 = full thickness cartilage defect [21, 22] (Fig. 2). The MRI measurements were performed by two other fellowship-trained musculoskeletal radiologists (Li and Yu, with 7 and 18 years of clinical experience, respectively) after all the data of the two groups were mixed and distributed blindly and randomly. All measurements were repeated by one of the radiologists (Li), and the interval between the first and second reading was at the last 4 weeks.

**Fig. 3:**
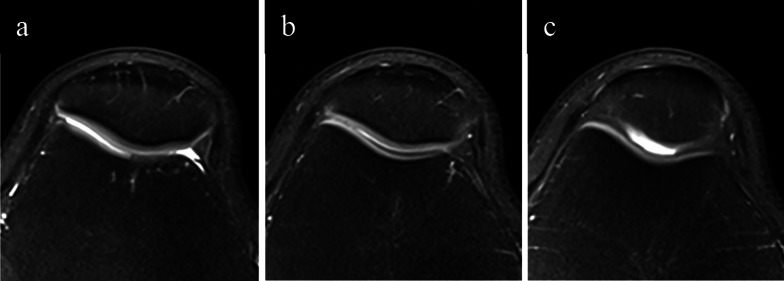
Illustration of the entrance plane of femoral trochlea, proximal slice and distal slice. **a** The proximal slice of the entrance plane of femoral trochlea, lateral articular surface showed the presence of cartilage, but the boundary was blurred, and medial articular surface was without cartilage covering. **b** The entrance plane of femoral trochlea, and it was the first craniocaudal plane where the complete cartilaginous trochlea can be seen. **c** The distal slice of the entrance plane of femoral trochlea, and the trochlea groove can be seen deepening

**Fig. 4:**
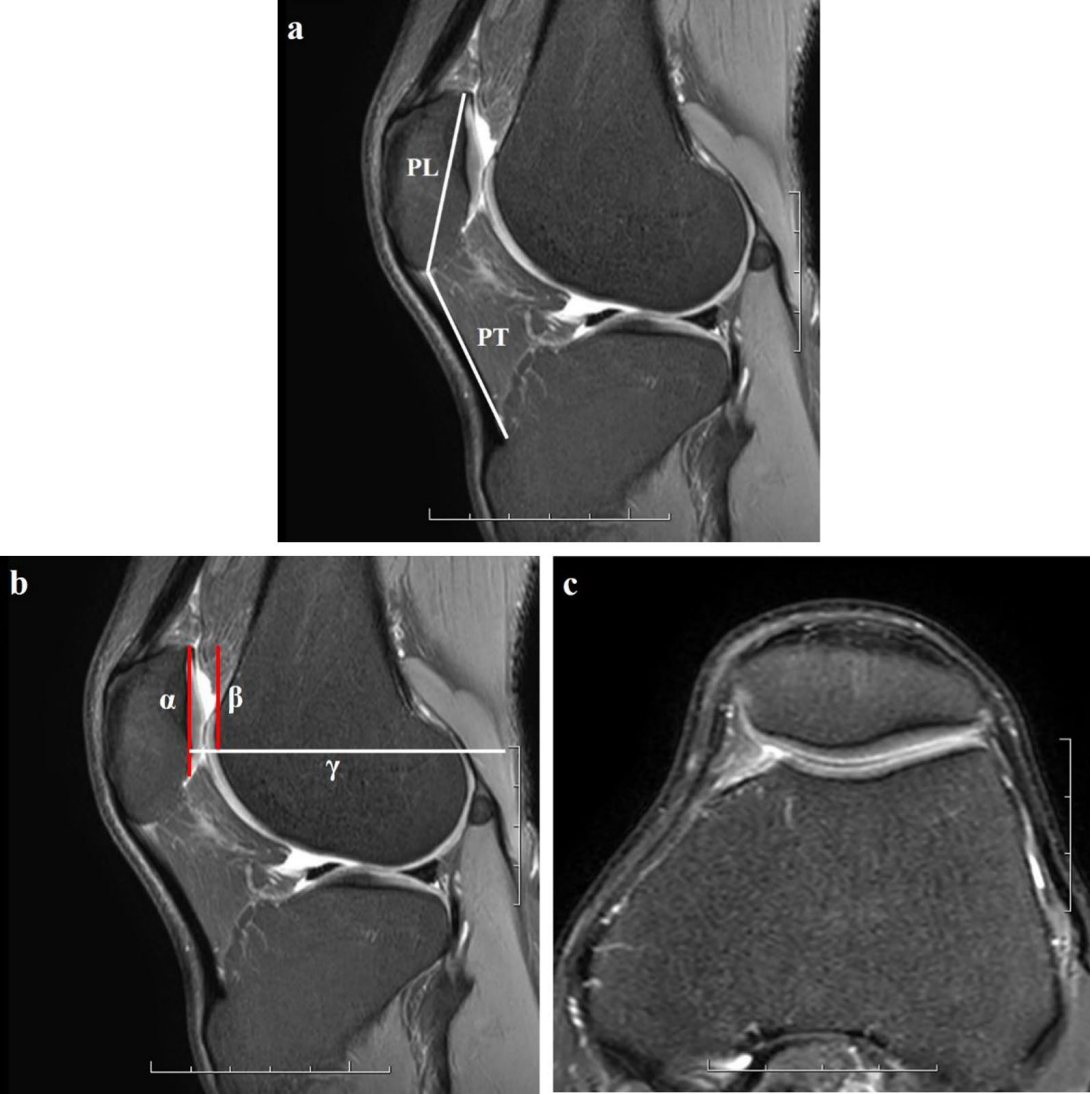
Methods for determining the Insall–Salvati ratio (**a**) and SP-ET index (**b**), demonstration of measurements of the related parameters on sagittal MR images. **a** The Insall–Salvati ratio was calculated as the patellar tendon length (PT) divided by the maximum patellar diagonal length (PL); **b** the SP-ET index was calculated as the distance between the most superior point of patella and the entrance of femoral trochlea (*β*) divided by the patellar articular surface length (*α*). Line *γ* was the reference line of the entrance plane of femoral trochlea; **c** the entrance plane of femoral trochlea, and it was the first craniocaudal plane where the complete cartilaginous trochlea can be seen

Two quantitative parameters for evaluating patellar position were assessed on the images of each MRI examination. The Insall–Salvati ratio was calculated as the patellar tendon length divided by the maximum patellar diagonal length on sagittal MRI image (Fig. 4a). The Insall–Salvati ratio was used to reflect the height of the patella, and the threshold was defined as > 1.2 [23]. The most superior point of patella-entrance of femoral trochlea distance ratio (SP-ET index) was used to reflect the vertical position of the patella relative to the trochlea. The entrance of femoral trochlea was the first craniocaudal plane where the complete cartilaginous trochlea can be seen [24, 25] (Fig. 3a–c). The sagittal plane with the greatest longitudinal diameter of the patella was used to obtain the SP-ET index which was calculated as the distance between the most superior point of patella and the entrance of femoral trochlea divided by the patellar articular surface length (Fig. 4b, c).

### Statistical tests

In this study, descriptive data were calculated for demographic data. The subjects were categorized, according to the inclusion and exclusion criteria, into the patellar cartilage lesions group and the control group. Intraclass correlation coefficients (ICCs) were used to assess the intra- and inter-reader reliability of MRI quantitative measures. Continuous variables with a normal distribution were expressed as mean ± standard deviation, and Student’s *t* test was used to detect the difference. The Spearman correlation was used to analyze the relationship between the two parameters. The above statistical analyses were performed using Windows software SPSS (version 17.0; SPSS, Chicago, IL, USA), and the significance level was set at 0.05. In addition, the diagnostic efficacy of two measurements of the patellar position was determined with receiver operating characteristic (ROC) curve analyses, which was performed using MedCalc statistical software (MedCalc Software, Mariakerke, Belgium). Optimum cutoff point, sensitivity, specificity, and area under the curve (AUC) of each parameter were calculated, and then ROC curves between the two groups were compared, and the significance level was set at 0.05.

## Results

### Demographic characteristics of the subjects in the two groups

During the study period, a total of 99 knees (25 left, 30 right, and 22 bilateral) of 77 patients (45 males and 32 females, age range 23–50 years, mean age 43 ± 7 years) were included in the patellar cartilage lesions group. There were 99 knees (51 left, 42 right, and 3 bilateral) of 96 participants (62 males and 34 females, age range 17–45 years, mean age 33 ± 8 years) in the control group (Table 1).

**Table 1:** Descriptive data

	Patellar cartilage lesions group (*n* = 99)	Control group (*n* = 99)
Mean age (years)	43 ± 7	33 ± 8
Gender (*n*, %)		
Male	32 (41.6%)	62 (64.6%)
Female	45 (58.4%)	34 (35.4%)
Side (*n*, %)		
Right	30 (39.0%)	42 (43.8%)
Left	25 (32.5%)	51 (53.1%)
Bilateral	22 (28.5%)	3 (3.1%)
Patella alta (*n*, %)	24 (24.2%)	–

### Intra- and inter-observer reliability of quantitative MRI measurements

The intra-reader ICCs for the SP-ET index and Insall–Salvati ratio were 0.930 and 0.875, respectively, and the inter-reader ICCs for the SP-ET index and Insall–Salvati ratio were 0.890 and 0.973, respectively, as shown in Table 2, indicating good intra- and inter-reader reliability.

**Table 2:** The intra- and inter-observer agreement between two radiologists of quantitative MRI measurements

Quantitative parameters	Intra-observer			Inter-observer		
	ICCs (95% CI)	*f* value	*p* value	ICCs (95% CI)	*f* value	*p* value
SP-ET index	0.930 (0.863–0.964)	14.088	< 0.001	0.890 (0.799–0.945)	8.855	< 0.001
Insall–Salvati ratio	0.875 (0.754–0.936)	7.846	< 0.001	0.973 (0.943–0.987)	38.141	< 0.001

*SP-ET index* the most superior point of patella-entrance of femoral trochlea distance ratio, *ICCs* intraclass correlation coefficients, *CI* confidence interval

### Comparison between the patellar cartilage lesions group and control group and the correlation between the two parameters

The two measurement parameters showed significant differences between patellar cartilage lesions group and control group. The Insall–Salvati ratio and SP-ET index in the patellar cartilage lesions group were significantly higher than those in the control group (*p* < 0.001) (Table 3). The SP-ET index showed a moderate positive correlation with the Insall–Salvati ratio (*r* = 0.307, *p* < 0.001).

**Table 3:** Comparison of MR quantitative parameters between the patellar cartilage lesions group and control group

	SP-ET index	Insall–Salvati ratio
Patellar cartilage lesions group	0.933 ± 0.115	1.100 ± 0.145
Control group	0.802 ± 0.108	1.004 ± 0.086
*t* value	8.227	5.709
*P* value	< 0.001	< 0.001

### ROC analyses of MRI quantitative measurements

**Fig. 5:**
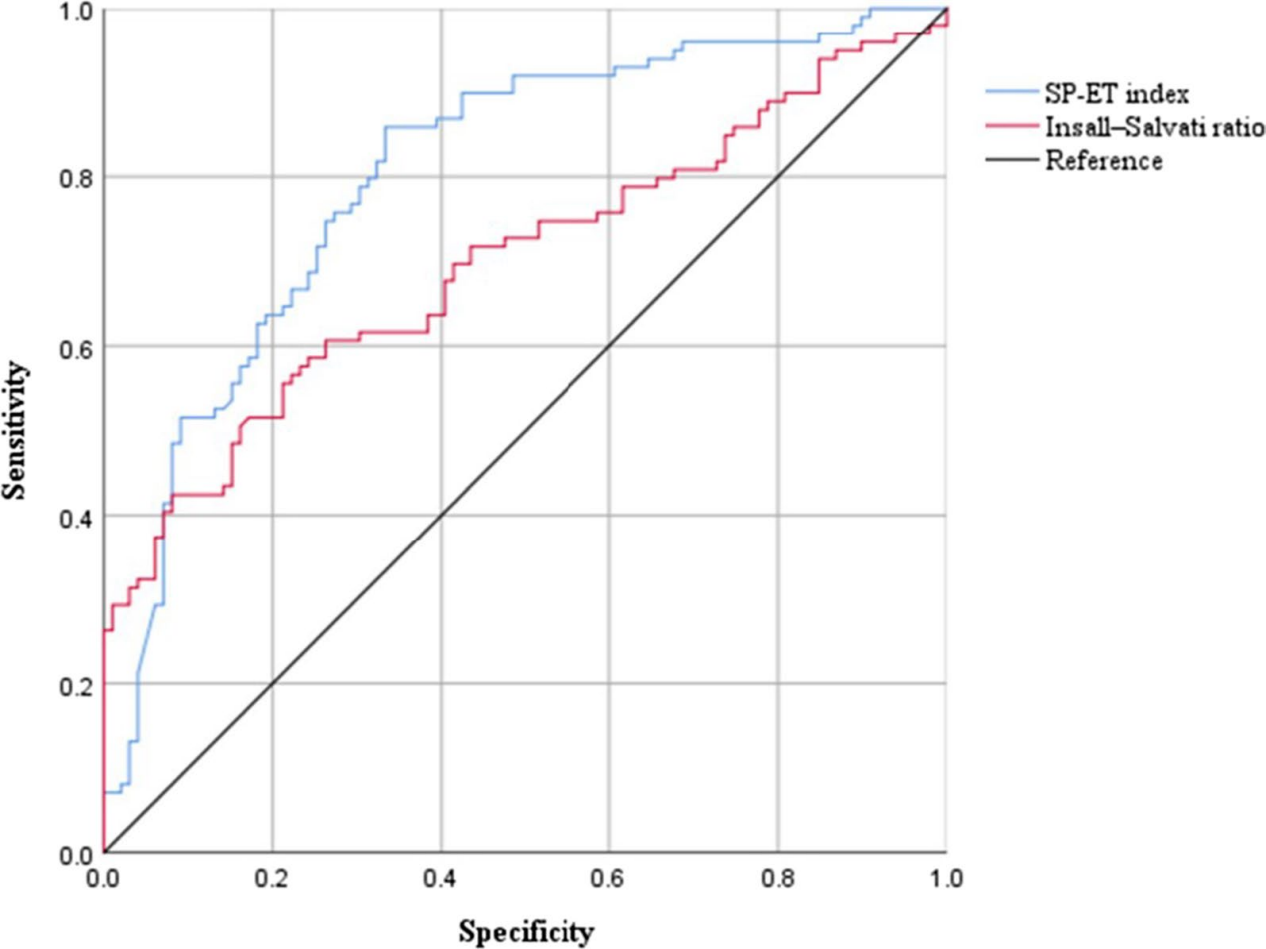
The receiver operating characteristic (ROC) curves of SP-ET index and Insall–Salvati ratio. The ROC of SP-ET index was the blue curve (area under curve (AUC) = 0.803); the ROC of Insall–Salvati ratio was the red curve (AUC = 0.699)

The ROC curves of the Insall–Salvati ratio and SP-ET index were generated (Table 4, Fig. 5). The area under the curve (AUC) of the SP-ET index was 0.803 (95% CI 0.740–0.856; Youden’s index 0.525; cutoff value 0.833; sensitivity and specificity were 0.859 and 0.667, respectively). The AUC of the Insall–Salvati ratio was 0.699 (95% confidence interval (CI) 0.629–0.762; Youden’s index 0.343; cutoff value 1.125; sensitivity and specificity were 0.424 and 0.911, respectively). The AUC of the SP-ET index was significantly higher than the AUC of the Insall–Salvati ratio (*p* = 0.027).

**Table 4:** The results of ROC analysis of the SP-ET index and Insall–Salvati ratio

	AUC (95%CI)	Abnormal MRI cutoff value
SP-ET index	0.803 (0.740–0.856)	> 0.833 (se = 0.859, sp = 0.667)
Insall–Salvati ratio	0.699 (0.629–0.762)	> 1.125 (se = 0.424, sp = 0.911)
*z* value	2.216	–
*p* value	0.027	–

*ROC* receiver operating characteristic

## Discussion

The main findings of the present study were twofold. Firstly, our data showed that increased SP-ET index was associated with the risk of developing patellar cartilage lesions. Secondly, the SP-ET index might be a better indicator to define the vertical position of the patella relative to femoral trochlear than the Insall–Salvati ratio in patellar cartilage lesions patients. These findings supported our hypothesis and indicated that the SP-ET index may be a useful complement parameter to define the vertical position of the patella relative to the femoral trochlear, and a higher position of patella might play a role in the pathogenesis of patellar cartilage lesions.

The previous researches have analyzed the influence of patellofemoral malalignment on patellar cartilage lesions or patellar cartilage defects [8, 11, 13, 15, 20, 26]. Overall, patellofemoral malalignment and structural abnormalities of the patellofemoral joint led to pathologic pressure on the patellofemoral joint might be an important pathogenesis of patellar cartilage lesions [20, 27]. Some studies found that a higher position of patella was an important influencing factor of patellar cartilage lesions [11, 15, 20], and in addition to the standard measurements of patellar height, some other measurements were analyzed, such as modified Insall–Salvati index [15, 20, 27], patellotrochlear index, and patellophyseal index [20, 27]. The assessment of the sagittal position of the patella is still worth discussing due to the diversity of examination techniques, including X-ray, CT, and MRI [5, 8]. Take the Insall–Salvati index, for example, which is an indicator of the height of the patellar and has traditionally been measured by conventional radiography [23]. But MRI is currently being used more clinically to assess the relationship between the patellofemoral joint. The advantages of MRI over conventional radiography lie in its capability of multiplanar, high-resolution imaging of chondral, and soft tissue lesions [1, 5, 8]. There have been some studies based on MRI measurements, but they are still controversial [20, 28]. Therefore, how to use MRI to accurately assess the sagittal position of the patella still needs to be discussed.

The Insall–Salvati ratio was defined as the length of the patellar tendon divided by the length of the patella, which was a validated and widely used index for evaluating the position of patella [23, 29]. The Insall–Salvati ratio can be measured on both lateral radiograph and sagittal MR image of the knee. A higher position of patella might result in failure of the patella to engage the trochlear groove early during knee flexion [17], and decrease the contact area between the patellar articular surface and trochlea [10, 16]. Previous studies have shown that patella alta was associated with patellofemoral joint pain, instability, chondromalacia, and osteoarthritis [1, 10, 30, 31]. In this study, though the Insall–Salvati ratio was significantly higher in the patellar cartilage lesions group than that in the control group, there were just 24.2% (24/99) of patients in the patellar cartilage lesions group showed a pathologic Insall–Salvati ratio of > 1.2, which coincided with the results of Ambra et al. [11]. Moreover, Ali et al. [28] found that the Insall–Salvati ratio did not correlate with the severity of patellotrochlear articular cartilage defected. In our opinion, the Insall–Salvati ratio in most patients with patellar cartilage lesions was in the normal range, which might reduce the instruction of this parameter in practical clinical work. Therefore, it was of great clinical impact to find more effective and direct evaluation indicators.

In the present study, the SP-ET index was used to define the position of the patella relative to the femoral trochlear, and SP-ET index showed a moderate correlation with the Insall–Salvati ratio. Ali et al. [28] and Mehl et al. [20] have used the patellophyseal index to assess patellar height in their studies, which was calculated as the distance from the most superior point of the patellar cartilage to the most superior point of the femoral cartilage divided by the length of patellar articular cartilage, and there were some similarities between the SP-ET index and patellophyseal index. However, the measurement of the SP-ET index only needed to be done at one plane (the sagittal plane with the greatest longitudinal diameter of the patella), and without the effect of axial displacement of patella. Therefore, we thought that the SP-ET index had a good practicability, and the intra- and inter-reader reliabilities of this parameter were also good in our study.

Mehl et al. [20] suggested that there was no significant difference in the patellophyseal index between the patellar cartilage defect group and control group. But different from the study of Mehl et al. [20], the study of Ali et al. [28] have found that there was a statistically significant difference in the patellophyseal index between normal group and severe patellar cartilage defect group. In the present study, we found that the SP-ET index in patellar cartilage lesions group was significantly higher than that in control group, and the AUC of the SP-ET index was significantly higher than that of the Insall–Salvati ratio. The difference in the results might be related to the different severity of the included participants in these studies, and the strict control of the flexion angle of the knee in our study. Because in our practical measurement process, it was found that the flexion angle of the knee had a great impact on the value of the SP-ET index. According to the results of these studies, we thought that the correlation of the SP-ET index and patellophyseal index with the occurrence and severity of patellar cartilage lesions/patellar cartilage defect required prospective and biomechanical studies to explore further.

Our study has several limitations. First, this was a retrospective study, and the selection of participants in the patellar cartilage lesions group was based on MR images without histologic or pathologic evidence. Second, the measurement errors might be caused by the formation of patella spurs, only patients with grade 1–2B patellar cartilage lesions were included in the patient group, therefore, patients with severe patellar cartilage lesions were excluded in this study. Third, the flexion angle of the knee has a great influence on the SP-ET index, so the measurement of the SP-ET index needs to control the flexion angle of the knee strictly. Fourth, our results showed that the specificity of SP-ET index was lower, which will increase the false negative rate, and may lead to an incorrect assessment of the sagittal position of the patella. Moreover, the comprehensive assessment of the influence of patellar and trochlear morphology on patellar cartilage lesions is also our next step. In addition, there were no strict age and gender matching between the two groups of participants. Future research efforts should focus on the relationship between the SP-ET index and patellofemoral osteoarthritis using a prospective longitudinal study, and the role of the SP-ET index in different grades of patellar cartilage lesions.

## Conclusions

The SP-ET index can be easily measured to define the vertical position of the patella relative to the femoral trochlear, and it showed a moderate correlation with the Insall–Salvati ratio which was a conventional measurement of patellar height. ROC analysis showed that the diagnostic efficiency of the SP-ET index was better than the Insall–Salvati ratio in patients with patellar cartilage lesions. Therefore, the SP-ET index showed promise as a useful complement parameter to define the vertical position of the patella, and a higher position of patella might be one of the factors in developing patellar cartilage lesions.

## Data Availability

The datasets used and analyzed during the current study are available from the corresponding author on reasonable request.

## Abbreviations

AUC: Area under the curve
CI: Confidence interval
ICCs: Intraclass correlation coefficients
MRI: Magnetic resonance imaging
PL: Patellar diagonal length
PT: Patellar tendon length
ROC: Receiver operating characteristic
SP-ET: Most superior point of patella-entrance of femoral trochlea distance
